# Establishment of a Quality Management System Based on ISO 9001 Standard in a Public Service Fungal Culture Collection

**DOI:** 10.3390/microorganisms4020021

**Published:** 2016-06-22

**Authors:** Marta F. Simões, Nicolina Dias, Cledir Santos, Nelson Lima

**Affiliations:** 1 CEB-Centre of Biological Engineering, Micoteca da Universidade do Minho, University of Minho, Campus de Gualtar, Braga 4710-057, Portugal; marta.simoes@kaust.edu.sa (M.F.S.); nidias@deb.uminho.pt (N.D.); 2 Computational Bioscience Research Center (CBRC), Computer, Electrical and Mathematical Sciences and Engineering Division (CEMSE), King Abdullah University of Science and Technology (KAUST), Thuwal 23955-6900, Saudi Arabia; 3 Department of Chemical Sciences and Natural Resources, Faculty of Engineering and Sciences, Universidad de La Frontera, Temuco 4811-230, Chile

**Keywords:** bioeconomy, customer satisfaction, microbial culture collection, microbiological resource centres, quality control

## Abstract

Collaborations between different Microbiological Resource Centres (mBRCs) and ethical sourcing practices are mandatory to guarantee biodiversity conservation, successful and sustainable preservation and fair share of benefits that arise from the use of genetic resources. Since microbial Culture Collections (CCs) are now engaged in meeting high quality operational standards, they are facing the challenge of establishing quality control criteria to certify their biological materials. The authentication/certification of strains is nowadays a demand from the bioeconomy sector for the global operation of mBRCs. The achievement of consistent quality assurance and trust within the mBRCs and microbial CCs context is a dynamic and never-ending process. A good option to facilitate that process is to implement a Quality Management System (QMS) based on the ISO 9001 standard. Here, we report a detailed description of all the steps taken for the QMS implementation at the Portuguese CC of filamentous fungi: Micoteca da Universidade do Minho (MUM). Our aim is to provide guidelines for the certification of other CCs, so that they can also enhance the search and choice of the most consistent, reliable, and effective operating methods, with assured procedures and validation of preservation; and guarantee trustworthy relations with all stakeholders.

## 1. Culture Collections and Biological Resource Centres

### 1.1. Background

Microbiological Resource Centres (mBRCs) represent the most advanced and up-to-date concept for Culture Collections (CCs) [1]. They are essential infrastructures, to preserve and manage the provision of the biological resources, to guarantee the microbial biodiversity conservation, and to successfully deliver benefits for Research & Development in different scientific fields [2,3,4,5,6,7,8,9,10].

The ever-growing pace of technology advance based on biotechnology worldwide, has lead to a continuous increase in the generation of large amounts of information and scientific knowledge (e.g., microbial genotypic and phenotypic information). As a result, CCs are responsible for storage, maintenance, and dissemination of these data [5,9]. Such responsibilities require a different approach in both material and data management and the adoption of new methodologies to ensure stability of procedures [11].

There are numerous types of CCs: national or regional, which supply samples and associated information; more localized CCs, that often support particular scientists; institutional CCs, that mostly provide internal institutional services, and the research CCs that provide services relevant to one or more research interests [5,9]. Regardless of having different origins, funding sources, and customers, and serving different purposes, public CCs (the ones that supply their products and services to the public, which will be the main focus of this manuscript) worldwide are now facing challenges related to their financial sustainability and quality control guaranty of the preserved microbial strains and associated information [12]. However, CCs can transition to mBRCs if they overcome their challenges and ensure financial sustainability, compliance with legislation, implementation of Quality Management Systems (QMSs), information and technology access, training and capacity building, taxonomic expertise, application of new technologies, and massive incorporation of biodiversity items [7,13,14].

The implementation of a QMS is the most important challenge to overcome because it is critical for quality control and quality assurance of microbial resources. Based on a QMS, CCs are able to supply microbiological materials with guaranteed harmonised international quality that complies with national and international legal frameworks (e.g., the Convention of Biological Diversity and, more recently, the Nagoya Protocol on access and benefit-sharing, quarantine lists, packaging and shipping regulations and permits, *etc.*) [7].

### 1.2. The Context of Quality Management System (QMS) and ISO Standards for CCs and mBRCs

The ISO 9001 standard, one of the best-known standards of ISO, was the first universally recognised management system standard available for many kinds of organisations (e.g., environmental, health and safety) [15,16]. This standard deals with the requirements that companies and organisations have to fulfil when they wish to voluntarily comply with the standard, and helps them to ensure that their products and services consistently satisfy their customer needs by maintaining regulatory compliance and focusing on continuous improvement [17,18].

The ISO 9001 certification is frequently used to establish confidence and improve relationships between organisations and their customers, this is why it is suitable to the improvement of CCs and mBRCs [19,20].

Effective implementation of a QMS implies complying with a Quality Policy (QP) created for the organisation, helping to attain defined quality objectives, and satisfying customers by presenting the demanded and required conform products and services [21,22]. However, defining quality objectives is not an easy task. It requires being aware of not only the current and future needs of the organisation and the markets, but also having a keen knowledge of all the necessary resources available, reaching results and drawing conclusions from a continuous quality evaluation. This evaluation, which is facilitated by the implemented QMS, is continuously assessed through regular meetings, management review, analysis of performance of products and processes, satisfaction level of stakeholders, benchmarking, and opportunities of improvement [22,23,24].

The ISO 9001 standard allows organisations to choose whether to include or exclude any given process from their QMS, and any changes to the QMS, identified as beneficial, can be introduced at any time because it is a flexible and dynamic system. It requires an external body to certify that an organization consistently provides quality products and services focusing on their continuous improvement as well as on customers’ satisfaction [23,25]. Overall, certified CCs, like any organisation, are able to be more structured and organised, qualify for more tenders and have more satisfied customers and, consequently, more orders and more profits.

Besides ISO 9001 standard, other standards can be applied to microbial laboratories [10]: the French standard NF S96-900 for the quality of BRCs, which is specific for BRC management systems and quality of the biological resources from human and microbiological sources [4,26]; the ISO/IEC 17025, for general requirements of competence for testing and calibration laboratories [27]; the ISO Guide 34, that specifies general requirements for the competence of reference material producers [26,28]; and the Brazilian standard NIT-DICLA-061 for the accreditation of both testing laboratories and those providers of biological reference materials in biological resource centres [29]. Because the ISO 9001 standard is harmonized and non-confrontational with the national and international standards presented above, it can be applied with other standards on any biological resource centre [22,30].

### 1.3. The Portuguese Fungal Culture Collection Micoteca da Universidade do Minho-MUM and Its Singular Characteristics

The Micoteca da Universidade do Minho (MUM) [31] is a fungal CC that was established in 1996. It is hosted in the Centre of Biological Engineering at University of Minho, Braga, Portugal. MUM collects, maintains and supplies fungal strains and their associated data, and also, acts as a centre of expertise, information and training in accordance with international quality standards, either for teaching and research in biotechnology as well as for life sciences.

MUM has developed a strong linkage with academia, industry, services, and other important stakeholders, contributing at national and international level to underpin CCs and related organizations, and close gap between science and society [32]. Furthermore, being established inside a university, has allowed MUM to become fully adapted to the academic atmosphere and access a good and common research infrastructure, within an international environment focused on research and entrepreneurship.

MUM actively interacts, cooperates and collaborates with other partners. It has joined the World Federation for Culture Collections—WFCC in 2001 with the registration number 816, being one of the six current Portuguese CCs (Table 1) registered in the World Data Centre for Microorganisms—WDCM. In 2002, MUM joined the European Culture Collections’ Organisation—ECCO, becoming member of the major European Organization of CCs. MUM was actively involved as a partner in the demonstration project of the Global Biological Resource Centre Network—GBRCN, and it was also involved on the European Consortium of Microbial Resource Centres—EMbaRC [9,33]. Moreover, MUM has been involved in the Microbial Resource Research infrastructure—MIRRI, a pan-European distributed infrastructure intended to encourage and facilitate innovation, research and development, in areas such as: pharmaceutics, biotechnology, healthcare and agriculture. MIRRI was envisioned to help deliver the Innovation Union goals, address societal challenges and support European Union competitiveness in the knowledge based bio-economy [9,34,35].

Because it was considered that MUM had all the parameters to guarantee customer satisfaction, it was a logical and natural step to aim for QMS certification.

### 1.4. MUM and Its Choice of ISO 9001 Standard for Quality Management

The quality assurance concept started to be embedded at MUM since its inception. MUM has always focused on meeting the needs of its customers by being influential, flexible, globally relevant, and attuned to international business and other organisations. This awareness became more evident due to the international panorama in which MUM is inserted and was the main catalyst that led to the implementation of a QMS based on the ISO 9001 standard.

The ISO 9001 certification was a choice made by MUM, in order to assure the strengthening of cooperation with other relevant CCs and also with industrial consortia, the scientific community and society in general. Considering the continuous improvement, MUM intends to significantly improve its processes, so that they become clearer, more transparent and rigorous to support the development of high quality deliverables. Moreover, a new edition of the ISO 9001 standard was published in September 2015, concluding over three years of revision work performed by experts from nearly 95 countries. The EN ISO 9001:2015 version reflects the current evolution of organizations operating multiple management systems, and focusing on the plan-do-check-act cycle and risk-based thinking [25]. Although MUM is already identifying risks (e.g., surveying the MUM’s interested parties and their relevant needs and expectations) and opportunities, changing the preventive plans into plans of actions, removing and updating some documents, and updating its QMS to fit the new version, the implementation of the QMS here described followed the quality principles and requirements of the previous version from 2008 [37].

This work explains the importance of Quality assessment and certification for CCs and BRCs—in guaranteeing their excellence regarding biodiversity conservation—and describes the step-by-step implementation of the QMS developed in the MUM CC to be used as a reference for the certification of other CCs.

## 2. The Quality Management System

### 2.1. Timeline and Tasks

Understanding the ISO 9001 standard is the beginning of all steps towards implementing a QMS. Consulting with outside organisations or individual entities can help to provide a clear understanding of the standard. For this reason, MUM decided to hire a local company for consulting services.

Besides being clear about all items referred to on the standard, being fully informed about everything related to the organisation is a must. MUM started by building a detailed knowledge of the collection including all aspects related to present and future intentions, services provided, location of operations and collaborators.

**Figure 1:**
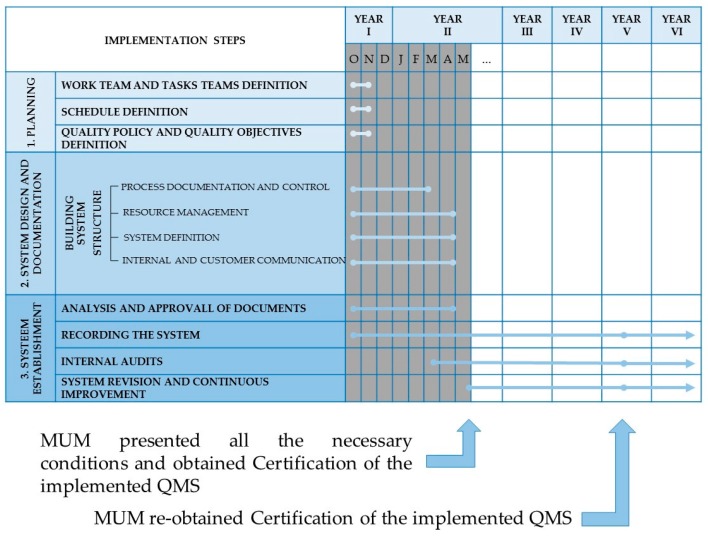
Timeline of the different phases from the implementation steps (grey area) until current state of the Quality Management System of the Micoteca da Universidade do Minho (MUM). The blue lines in bold represent the duration of the item during the represented timeline. The grey columns represent the time previous to certification. O, N, D, J, F, M, A and, M, under year I and year II, correspond to the months.

Once all the details and specifications necessary to achieve the final product were defined, a conformity assessment of the available arrangements for quality management was made. Then, a set schedule presented in a detailed timeline (grey area in Figure 1), with all the required and listed actions, was established and followed.

The QMS implementation lasted almost one year, during which three main steps were developed.

#### 2.1.1. First Step of Implementation: Planning

The first step was the planning of the QMS, where specific tasks were attributed to the collaborators, deadlines were established and a QP as well as Quality Objectives were defined. The commitment to quality, the value of costumer needs and the supply of high quality products and services were taken into account to define the QP. A vision and a mission were also defined and the key processes—Material Reception Process (MRP), Material Preservation Process (MPP) and Material Supply Process (MSP)—were established. Besides the infrastructures, which are critical for the QMS, the defined processes are exclusive and for dedicated use of the collection.

#### 2.1.2. Second Step of Implementation: System Design and Documentation

The second step of implementation was to design the system and create the required QMS documentation. Also, a documented procedure was elaborated in order to rapidly track back the latest version of any QMS document and to comply with the requirement of control of documents/records of the standard. Another procedure was created for the control of documents stipulating all details regarding identification of documents, file mode, retention period and manner of destruction. All electronic documents are to be subjected to regular backups on a weekly basis on the server with dedicated and exclusive use for the collection, and other documents kept in paper format are to be properly stored. All documents will have a retention time of five-years, during which if updated or considered obsolete will be replaced and/or destroyed or deleted. Specific records made in laboratory notebooks with numbered pages are to be kept forever in the archives of MUM.

In addition to the most important document of the QMS, the Quality Policy (QP), other activities and documents were created, namely: (1) description and implementation of processes, procedures and standard operating procedures (SOPs); (2) selection, evaluation and qualification of suppliers through the analyses of related non-conformities, records and corrective actions; (3) infrastructure and work environment maintenance records; (4) definition of measurement and monitoring equipment with established inventory, equipment charts, control and maintenance plan; (5) human resources management with defined minimal skill, responsibilities and job description; and (6) customer’s satisfaction evaluation through satisfaction inquiry, complaints evaluation, suggestions and quality plan.

In the strategic plan of MUM for the following years, a commitment to six self-defined general objectives (Table 2) was taken.

The (also self-defined) mission statement of MUM, just as the objectives, intended to address global challenges along with customer needs. Being action-oriented and in order to attain its vision and mission, MUM set several operational objectives (Table 3), which, together with a series of specific actions for their accomplishment, establish the strategic plan of action.

The general and operational objectives of MUM (Table 2 and Table 3, respectively), were formulated in accordance with the established QP, and as the key performance indicators (KPIs), they are to be revised annually. They were developed in compliance with recognised best practice and objectives, from other types of standards or specifications such as the Organisation for Economic Co-operation and Development (OECD) guidelines for BRCs [3].

#### 2.1.3. Third Step of Implementation: System Establishment

The third and last step was the establishment of the QMS with the achievement of certification. From this date on, MUM has been keeping and will keep on performing a QMS under constant revision, as well as annual internal and external audits to identify issues, guarantee compliance and to continuously improve. This will be ensured by: audits, efficacy analysis, corrective and preventive actions, planning actions, and annual management reviews of the entire system. Besides, as required by the ISO 9001 standard, documentation and records will provide evidence of compliance.

The real advantages of implementing a QMS begin after the certification process, when all the collaborators are already engaged with the system and all the processes are done according to the created specifications. Nevertheless, proper and continual maintenance of the system is required to detect any inconsistencies and assure continuous improvement. Keeping records allows for provision of information on the adherence to system practices, as well as on the groundwork for process control measurement. And as stated on the standard [38], the revision of all measurements and the response to observed tendencies assure the desirable continuous improvement of the system.

## 3. Results

### 3.1. Planning of the Quality Management System

The designed timetable proposed a detailed agenda, covering most issues and topics during the planning, which facilitated compliance to the entire system.

In the QP, a set of MUM’s intentions and orientations was formally expressed, conveying the commitment to Quality by valuing costumer needs and supplying high quality products and services. To warrant the QP, a main goal was established—the on-going development of management processes to continuously improve the service. This goal was achieved by: using and developing KPIs; creating a dynamic, innovative and high-quality work environment; following the technological and scientific development of the sector; and continuously developing the expertise, professionalism and integrity of all collaborators.

MUM’s QP constitutes the baseline reference of its QMS, defining the general objectives from the perspective of requirements to be met and continuous improvement. In order to verify its constant adequacy, it was considered important to establish an annual revision of the statements, and to ensure that the QP is properly conveyed, understood and interiorised by all the collaborators of MUM and it is disclosed outside the collection (e.g., available on the website of MUM [31]).

MUM defined its vision as “a world in which fungal diversity is preserved and available for all” and its mission as “to be a resource centre for fungal biodiversity preservation and information, and create solutions for sustainable development and human well-being”. It also defined three processes for: reception (MRP), preservation (MPP) and supply of materials (MSP). Each process was fully described and schematized with inclusion of all related actions. All steps were detailed and all the forms and standard operating procedures (SOPs) to be used in each step of the process were included on each scheme and all the documents for the QMS were created and organized.

### 3.2. System Design and QMS Documentation

Designing the system and creating all the documents for that system are two tasks that are adapted to the needs of the organisation implementing its QMS. And, in order to provide evidence of revisions and internal distribution of documents, a specific form was created to be signed as a record of each revision, update, alteration or distribution.

The documentation of the QMS consists of two types of documents: external and internal. External documents include recommendations and disclosure documents both legal and scientific that serve as references for the processes (MRP, MPP and MSP), procedures, and the entire QMS documentation. All collaborators and a consultant met on a regular basis to decide on which documents to be used according to MUM’s objectives.

The internal documents comprise all the documents that are directly related to the processes and all tasks described in them must be performed exactly as they stipulate for they imply compliance to what has been described as essential to the processes.

**Figure 2:**
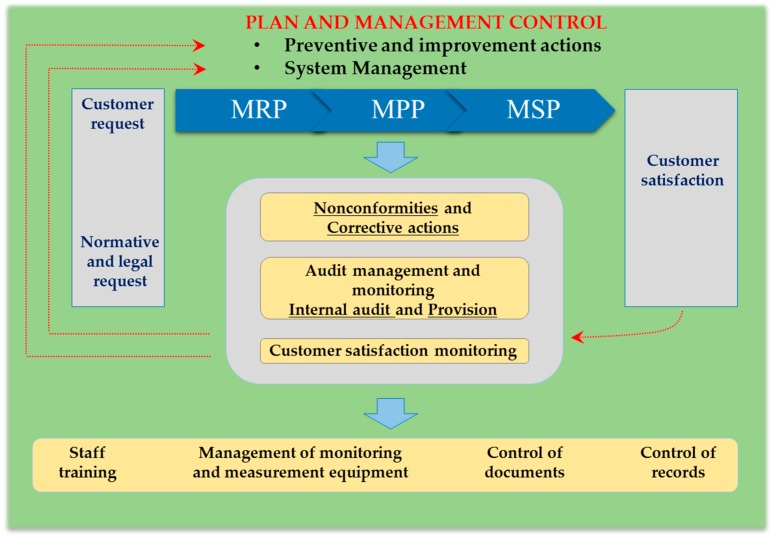
Model of key system areas of ISO 9001 implemented at Micoteca da Universidade do Minho. Being MRP, MPP and MSP the defined processes; and all contents of yellowish orange boxes the defined procedures.

The processes, which sustain the QMS and their interrelationships, are illustrated in the model of key system areas of ISO 9001 designed for MUM (Figure 2). They state the responsible person for performing the activities, the criteria for decision-making and for the methods that assure the performance of these activities in effective and efficient terms, the necessary information for their realisation, their objectives and their applicable indicators.

In the description of the processes, the referred indicators are identified as likely-to-be-used for continuous monitoring of these processes. However, their effective use depends on the defined operational objectives for each period (Table 3).

Furthermore, for all the procedures (namely: Nonconformities and corrective actions, Control of documents, Control of records, Management of monitoring and measuring equipment, Preventive and improvement actions, System management, Staff training, Criteria for deposit of strains, Internal audit, Provision and Control of access), a set of information—objectives, scope, responsibilities and detailed information on how to proceed—was established, defined and recorded.

Analysing the level of performance success and the reach of quality objectives is part of the system design and is done through the use of quantifiable parameters—KPIs—defined for this QMS as follows: (1) percentage of strains mistakenly identified or with insufficient information; (2) percentage of requests accepted for deposit; (3) percentage of non-viable strains after accepted request for supply; (4) percentage of complaints; (5) general index of customer satisfaction (average obtained from the answers to a satisfaction inquiry); and (6) time elapsed between request and response to that request. However, the KPIs are self-defined by MUM (or any organisation that decides to pursue certification according to ISO 9001) in order to be possible to evaluate and monitor the processes of the implemented system, and their effective use for each period being revised during the audits, depends on the defined objectives. With the evolution of the QMS, and the changes of the objectives that occur from the continuous improvement imposed by the ISO 9001 standard, other indicators might be identified and created for the processes adding or replacing the existing ones.

### 3.3. Quality Manual and Manual of Functions

The Quality Manual, which defines the entire QMS, is to be applied in all the functional structure of MUM. It describes all the procedures adopted by MUM to ensure a QMS adequate to its activities, and it includes the organisational structure, responsibilities, practices, procedures, processes and resources. It also presents the QP that includes the mission and reflects the set of chosen standards. Even though the Quality Manual is no longer an ISO 9001 standard requirement, it adds value to the QMS and its scope and interactions between the processes still need to be defined [39].

Functions for the collaborators of MUM were stipulated and established, and an organigram was defined and included on the Quality Manual. For MUM, being a small CC with about 500 strains available in the e-catalogue, the functions specified were: Director, Quality Director, Quality Manager, Curator, Technician and Internal Auditor (only hired at specifc times for internal audits). The functios of Director and Quality Director are attributed to one person only, with a 1.0 full-time equivalent (FTE). The curator works full-time (1.0 FTE) and the technician and the Quality Manager work part-time (0.6 FTE). The Manual of Functions stipulates the qualifications and job description for each function. For example, it is the responsibility of the Director to ensure compliance at all levels, of all the determinations set forth in the Quality Manual and, being the higher rank, he must be committed to all his attributed duties.

The Quality Manual also defines the best practices adopted at MUM and states that it must identify physical resources and infrastructures necessary for its proper function and operation. The infrastructure that supports the processes is adequate for the production of the product and the supply of services, and includes: buildings, equipment, furniture, air-conditioning system, informatics hardware and software, and communications network. To maintain the infrastructure, preventive and corrective maintenances are required. The preventive maintenance is planned and then registered after being performed. This is how the working environment is maintained, specifically the environmental conditions, the air conditioning system and systems of incubation, refrigeration and deep-freezing. Furthermore, and given their relevance to the system, the refrigeration and deep-freezing are measured and monitored by specific equipment (data logger) to assure the proper preservation of samples.

### 3.4. Analysis and Approval of Documents

MUM assumed and described on its Quality Manual, the compromise of assuring annual revision of its QMS in order to verify its adequacy. During this meeting for revision, all data derived from the system must be analysed and properly treated. Any documents found to be unsuitable for the QMS are altered into new versions or removed from the system. With time, new forms can be created if collaborators find it necessary. The performance of the QMS must be evaluated and future actions to improve the overall performance must be defined at this time, all evidenced in minutes from the meeting.

Regular records, documented on appropriate forms, of daily routines defined on the processes generate evidence of the implemented QMS and guarantee traceability for each step of each process.

By complying with the established procedure for monitoring of general and associated documents, MUM ensures that the basic documents supporting QMS are: (1) defined in terms of format and content; (2) approved before being issued; (3) subjected to review and update when necessary, making it possible to identify their review status at any time; (4) available, in an updated version, in all the locations where they are needed; (5) kept legible and available for use during a period of time appropriate to the needs of MUM and to its commitments with customers and other stakeholders; (6) available in informatics or physical folders and; (7) whenever becoming obsolete, promptly removed from the daily use, destroyed or identified as obsolete.

According to the dispositions described on the procedure for the control of records, MUM assures that the records that evidence the functioning of the QMS and the obtained results are controlled for their unambiguous identification; kept in appropriate storage conditions; protected from damage caused by misuse; kept for a period of time compatible with the commitments of MUM with the customers and other stakeholders; and, destroyed appropriately to prevent improper dissemination of information with confidential character or with exclusive interest for MUM.

The QMS is regularly analysed and annual Internal Audits are performed to assess the effectiveness of the QMS and evaluate the compliance with the requirements of processes and reference standards. Internal Audits are made in order to specifically analyse: adequacy of the QP; Quality Manual; Quality Objectives and other system documentation; previous audit results; feedback of customers; results of monitoring and measuring processes, product conformity and quality objectives; status of corrective actions and preventive actions; follow-up actions from previous management reviews; changes that might affect the QMS, including legislation and regulatory changes; and defined recommendations for improvement.

### 3.5. Achievement of Certificates

In Portugal, the Associação Portuguesa de Certificação (APCER) is the only Portuguese entity representative of the International Certification Network (IQNet) which allows immediate international recognition of entities certified. To issue a certification to an organisation, APCER first audits the organisation.

The audit for concession of certification was performed at MUM by an auditor from APCER. As in all posterior audits, all elements of the ISO standard were covered. The auditor looked for evidence of compliance with the ISO standard and with internal documents. This evidence included: records of analysis, customer contracts, equipment and calibration reports, training records and, interviews of personnel. Any non-conformity detected had posterior corrective actions, described in appropriate forms which were reviewed by the auditor on a following audit.

**Figure 3:**
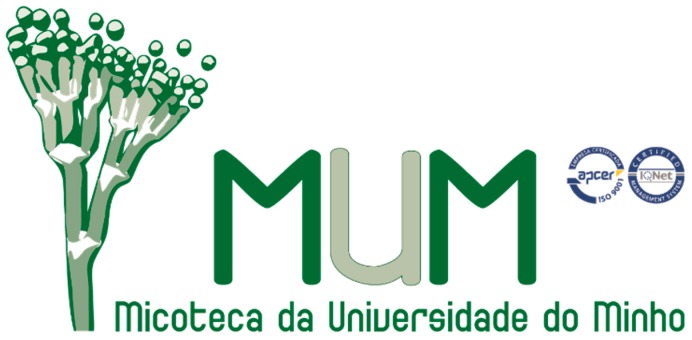
Logo of certified organisation by Associação Portuguesa de Certificação (APCER).

After the initial audits, MUM obtained its certification and received the certificate of registration proving conformity and the international certificate from IQNet. From this date on, MUM started to include the certification logo (Figure 3) on marketing and anouncements documents and emails. The use of the logo came with some rules that were included on the list of External documents.

About a year after obtaining certification, MUM performed a new Internal Audit and a new first follow-up audit was performed from APCER where it was checked that the QMS provided all the evidences of its maintenance and continuous improvement.

From this point, regular annual revisions were made to the QMS followed by internal and external audits. Three years after the initial certification, MUM re-obtained certification from APCER, confirming the health of the implemented QMS and its continuous improvement.

## 4. Discussion

### 4.1. ISO 9001 at Micoteca da Universidade do Minho

Regarding CCs context, appropriate access to resources requires not only a fair access policy but also a QP that can be implemented via a QMS. This is why MUM decided to achieve consistency in products management by identifying and implementing a suitable QMS with external certification based on the ISO 9001 standard, selected because of its wide scope and generic character. The QMS for MUM was designed according to the specific and changing needs of the collection. Whenever any requirements of the standard cannot be applied, due to the nature of an organisation and its product, these can be considered for exclusion. MUM considered the requirements for “Design and Development” as excluded—clause 7.3 from the 2008 version of the standard—because they were deemed as not applicable to its activity. The exclusion of this clause was considered not to affect the ability nor the responsibility to provide products and services that satisfy customer requirements, and legal frameworks. However, it should be noted that MUM has all the infrastructures to change this exclusion and include it on the implemented QMS. MUM has evolved from an internal quality to a QMS with international recognition and aims to extend the scope of the certification to include R&D and training since training and education services are also provided by many CCs [40].

The cost of certification is ultimately determined by the size of a CC, the number of collaborators working within it, the financing support CCs have (institutional, governmental or private), and the different values that change from country to country. In order to prepare for certification, which can include consulting services, acquisition of new equipment (e.g., data loggers, freeze-driers or power supply units) and updating of others already being used (e.g., maintenance or calibrations), 20,000 euros were spent at MUM. Part of the certification cost is fixed and include a guaranteed fixed fee for certification. At MUM, a regular and annual cost of 3000 euros has been spent on audits, maintenance and calibrations. Concerning the man-power due the certification process an increase of 0.8 EFT was estimated with a correspondent cost of 10,000 euros per year. An implemented QMS needs over time investments for improving processes, infrastructure and the collection as a whole and this can be a critical point for some collections.

Competitiveness is constant nowadays, not only from the point of view of supply capacity to the customer, but also in terms of support/finance/attribution of projects and others in terms of finances. There is always the need to improve internal operations, validate the know-how, and qualify all the involved collaborators. The implementation of a QMS and its certification highlight the will and action of organisations that take into account all of the above and the expectations of both customers and stakeholders.

It is noteworthy to mention that for most strains, the first preservation upon reception was prior to the implementation of the QMS. The procedures developed at that time were not under the now-implemented system. But from the date of implementation, whenever one of these strains undergoes the actions described in any process, all details are followed according to what was established. All strains available in e-catalogue have all traceable information related to them perfectly recorded and updated on the collection’s database, and the QMS guarantees that all future preservations and manipulations of either new or already preserved strains will follow the described processes, their associated procedures and all detailed actions. Therefore, a pre-defined price is charged to the customer (values, available upon first contact made with MUM, at the current date are: 50 euros per strain supplied and 250 euros for confidential strain deposited, these values do not include the VAT rate).

The ISO 9001 allowed MUM, and allows all other CCs that voluntarily opt for implementing a QMS based on ISO 9001, to become part of a socio-economic logic that combines high-level scientific research with the provision of rigorously managed specialized services.

This is also the logic behind the concept of mBRC that intends for traditional CCs to evolve into better support infrastructures for science and biotechnology.

### 4.2. Micoteca da Universidade do Minho before and after Its Quality Management System

Since its beginning, MUM always aimed for better quality but initial efforts were very cumbersome and demanding and only presented long-term results. The implementation of a QMS based on the ISO 9001 with the active contribution of all collaborators led to a more committed team where all contributed with their inputs to make the QMS more effective. MUM reached a new step in its existence, attaining all the necessary conditions for the certification of the implemented QMS and obtaining the certificate of ISO 9001. Certification was obtained for Deposit, Preservation and Supply of Filamentous Fungi, from the independent assessment agency APCER with the International Certification Network—IQNet. With this, MUM granted itself a more distinguishable spot among its partners, providing greater credibility and competitiveness nationally and internationally. Of the 707 worldly scattered CCs from 72 countries registered in the WDCM [41], MUM is the 1st Portuguese and the 23rd CC in the world to obtain this qualification.

Certification was the result of implementing an effective quality system that now allows: faster identification and resolution of any steps or parameters regarding methods, collaborators or equipment; improved customer satisfaction, achievement of quality requirements, and overall increased quality of services. It is important to note that after implementing the QMS and keeping the maintenance of all its key elements, the work is not finished. Indeed, it is an on-going task and there is still a beneficial obligation of performing regular Internal Audits to keep on achieving continuous improvements. There are specific advantages from the QMS implementation and certification, noted by MUM and its collaborators, these being: improved control and planning, improved efficiency and productivity, consistency in products and services, reduced waste of time and resources, reduced costs and, improved collaborators with higher retention capability and higher motivation.

MUM has now a clearly-defined vision and mission and benefits from experienced collaborators; possesses well-preserved and characterised filamentous fungi supported by an excellent database; is a descriptor of species new to science; leads Portuguese studies of filamentous fungi; is internationally recognised as a structured CC of reference in Portugal; is a core member of international initiatives and projects; is well known for very high numbers of international research papers, post-graduate research, and R&D projects; and, has excellent relationships with stakeholders and end-users to sustain its work. The implemented QMS facilitated all of the above and turned certification into a natural evolution of the collection. Furthermore, certification has allowed MUM to have a better international status and move towards becoming a mBRC as defined by OECD. All the referred data prove the high level of commitment of MUM to keep striving for continuous improvement and grant MUM with a higher demand of products and services from national and international customers that has been increasing since the implementation of the QMS.

Faster detection and resolution of issues regarding methods, collaborators or equipment, improved customer satisfaction, and overall increased laboratory activities are all the result of implementing a functional and successful quality system. MUM intends to keep its system sharp and continue the follow-up of the already obtained certification.

Quality control is a must to ensure that CCs provide authenticated products and have the best services. To better assure and maintain the search for excellence, MUM is also considering to do its accreditation for critical assays (e.g., sterility, purity, viability and identity), and will be attentive to new standards that might be developed and applied to mBRCs. Accreditation, just like certification, is not mandatory but it adds another level of confidence, as ‘accredited’ means the certification body has been independently checked to make sure it operates according to international standards [27]. It is usually limited to certain procedures or services rather than applied to all operations [9]. The quality of MUM can be even more assured by complementing the already existent certification with a future accreditation (e.g., based on the standard ISO/IEC 17025 or the new standard involving general requirements for biobanks that is developing on the ISO technical commission 276 for biotechnology). MUM would then benefit from more frequent audits by external auditors to keep the system sharp and continue the follow-up of the already obtained certification.

## 5. Conclusions

Globalisation has made the world smaller, making the challenges of few into the challenges of many, and leading to increased expectations from customers and other interested parties. Culture collections worldwide have been gathering efforts to develop standardized guidelines applicable for their products and services. This has been done through projects like the previously mentioned EMbaRC or MIRRI as well as with the help of institutions like ECCO, WFCC and OECD. This publication is intended to present MUM as a case-study that shows how its ISO 9001-based QMS was implemented with the outcome of independent certification and to facilitate this process for other CC.

After achieving ISO 9001 certification at MUM, it has been easier to detect and correct gaps and flaws in all methods and procedures, and trace all essential items regarding the final products which lead to substantially upgraded quality of services and products. More stringent quality parameters have been introduced to identify and authenticate biological material preserve at MUM.

We realised that having clearly-defined objectives and effective actions helps to maintain the core values and achieving the vision and mission of the CC. Furthermore, we stress the importance of achieving collections certification to recognise the value of their activities.

## Figures and Tables

**Table 1:** Portuguese culture collections registered at the World Data Centre for Microorganisms (Adapted from: [36]).

WDCM No.	Acronym	Collection
WDCM 595/1069	IGC/PYCC	Portuguese Yeast Culture Collection
WDCM 761	CCMI	Culture Collection of Industrial Microorganisms
WDCM 816	MUM	Micoteca da Universidade do Minho
WDCM 881	MEAN	Micoteca da Estação Agronómica Nacional
WDCM 906	ACOI	Algoteca de Coimbra
WDCM 1089	LEGE	Blue Biotechnology and Ecotoxicology Culture Collection

**Table 2:** List of general quality objectives of MUM.

No.	Description
1	MUM deliverables’ will meet customer needs
2	MUM standards’ will promote innovation and provide solutions to address global challenges
3	MUM will excel in reaching out to and engaging stakeholders
4	MUM will foster partnerships that further increase the value and efficient development of international standards
5	MUM and its processes will be significantly improved
6	MUM and the value of international standards will be clearly understood by customers, stakeholders and general public

**Table 3:** List of specific operational quality objectives.

No.	Description
1	Strive for complete understanding and assurance of the needs of customers.
2	Obtain a general customer satisfaction index of a minimum acceptable value and a participation of a minimum number of the customers.
3	Measure and monitor processes through a set of a minimum number of indicators per process.
4	Follow the technological and scientific development of the sector.
5	Continuously develop the expertise, integrity and professionalism by performing a minimum of training hours per collaborator.
6	Maintain a dynamic team, perform high-quality research, and follow the technological and scientific development of the sector.
7	Act in a preventive manner identifying, at least, four preventive or improvement actions per year.

## Abbreviations

The following abbreviations are used in this manuscript:

mBRC	microbiological resource centre
ISO	International Organisation for Standardisation
KPIs	key performance indicators
MUM	Micoteca da Universidade do Minho
QMS	Quality Management System
SOP	Standard Operating Procedure
CC	Culture Collection
QP	Quality Policy
